# Effects of diet and fitness apps on eating disorder behaviours: qualitative study

**DOI:** 10.1192/bjo.2021.1011

**Published:** 2021-09-24

**Authors:** Elizabeth V. Eikey

**Affiliations:** Herbert Wertheim School of Public Health and Human Longevity Science and The Design Lab, University of California, San Diego, USA; Information Sciences and Technology, Pennsylvania State University, USA

**Keywords:** diet and fitness app, calorie counting, weight loss, unintended consequences, self-tracking

## Abstract

**Background:**

Diet and fitness apps are often promoted in university and college settings and touted as a means to improve health with little attention given to unanticipated negative effects, especially among those at risk for or with eating disorders.

**Aims:**

Few researchers have studied how these apps affect women with eating disorders in university and college settings. This research investigates the unintended negative consequences of engaging with these tools.

**Method:**

Data collection sessions comprised three components conducted with 24 participants: survey (demographic and eating disorder symptoms), think-aloud exercise and semi-structured interview. Thematic analysis was used to analyse data.

**Results:**

Participants reported that diet and fitness apps trigger and exacerbate symptoms by focusing heavily on quantification, promoting overuse and providing certain types of feedback. Eight themes of negative consequences emerged: fixation on numbers, rigid diet, obsession, app dependency, high sense of achievement, extreme negative emotions, motivation from ‘negative’ messages, and excess competition. Although these themes were common when users’ focus was to lose weight or eat less, they were also prevalent when users wanted to focus explicitly on eating disorder recovery.

**Conclusions:**

Unintended negative consequences are linked to the quantified self movement, conception of appropriate usage, and visual cues and feedback. This paper critically examines diet and fitness app design and discusses implications for designers, educators and clinicians. Ultimately, this research emphasises the need for a fundamental shift in how diet and fitness apps promote health, with mental health at the forefront.

Diet and fitness apps often are touted as a means to improve users’ health. Most of these apps consist of nutrition, food, physical activity, weight and even sometimes body measurement tracking tools and connections to a community of users with similar goals. Although these apps are popular^1,2^ and can be helpful to some,^3^ they can have unintended adverse effects on others, such as university and college students.^4–7^ These apps often overwhelmingly focus on weight loss and normalise weight control methods. Using weight loss as a proxy for health is problematic because it may increase the risk of or exacerbate eating disorder behaviours in already susceptible groups, such as women attending university.^8^ Further, these apps tend to overlook the role of mental health in addressing physical health challenges. The emphasis on weight loss within these apps is consistent with and feeds into Western cultures’ obsession with thinness and dieting. Diet and fitness apps also support and encourage dieting behaviours. This is an issue because dieting behaviours and unhealthy weight control methods are also risk factors for eating disorders.^9,10^ Additionally, when compared with, for instance, paper tracking, these apps are always on hand and more discreet because of the prevalence of and norms around smartphone use, making it easier to constantly engage with diet and fitness content. They also often provide users with a set plan based on how much weight they want to lose, and leverage features, such as progress visualisations to influence behaviour, colour choices to denote positive and negative behaviours, and reminders and streaks to encourage consistent tracking. Although disordered eating affects all genders, eating disorders and eating disorder-related behaviours are extremely prevalent among women, especially those in college and university settings.^8,11–13^ In fact, researchers have found that 13.5% of undergraduate women screen positive for eating disorders,^8^ and 40–49% of university women engage in eating disorder behaviours at least once a week.^13^

Only somewhat recently have researchers studied diet and fitness apps in the context of eating disorders.^4–6^ For example, Honary et al^6^ found that almost half of the young people who participated in their study had maladaptive eating and exercise behaviours from using diet and fitness apps. In their study of 493 college students, Simpson and Mazzeo^4^ found that those who reported using diet and fitness apps had higher levels of eating disorder symptoms. Similarly, but focusing on individuals with clinically diagnosed eating disorders, Levinson et al^5^ found that in their cohort, 73% of those who used MyFitnessPal perceived it as contributing to their eating disorder, and these perceptions were correlated with eating disorder symptoms. Although these studies are important to recognise the link between eating disorders and diet and fitness apps, they do not shed light on how these apps may unintentionally affect users and their eating disorder symptoms. To address this gap, a qualitative study was conducted to answer the following research question: what are the unintended negative consequences of diet and fitness apps among women attending university who exhibit eating disorder-related behaviours? The term ‘unintended consequences’ refers to unforeseen or unpredicted results.^42^ This terminology is common when discussing technological impact, especially related to health information technology. These consequences can be positive, negative or neutral, but often refer to adverse effects.

## The research question

This study takes an interpretivist perspective: knowledge is contextual and grounded in participants experiences.^15^ This paper reports on one portion of a study on the use, impact and perceptions of diet and fitness apps (and if they are used in conjunction with other technologies, such as social media). Eight themes emerged that highlight the unintended negative consequences of diet and fitness apps. Findings from this study can be used by app designers, educators and clinicians to more carefully consider how these apps affect users, especially young women to whom these apps are often marketed.

## Eating disorder behaviours

For the purposes of this research, eating disorder behaviours are behaviours associated with anorexia and bulimia nervosa. These include excessive calorie or food restriction; intense fear of gaining weight; obsession with weight and consistent behaviour to prevent weight gain; self-esteem overly related to body image; bingeing; feeling of being out of control during bingeing; purging; dramatic weight loss; preoccupation with weight, food, calories, fat grams and dieting; refusal to eat certain foods; comments about feeling ‘fat’; hunger denial; excessive exercise regimen and development of food rituals.^16^ Because many women do not see a professional for their symptoms and thus never receive a diagnosis,^8^ eating disorder behaviours in this context may or may not indicate full clinical eating disorders or qualify to be categorised as other eating disorders, such as other specified feeding and eating disorder or unspecified feeding and eating disorder. The women in this study self-identify as having an eating disorder. Therefore, in the remainder of this paper, eating disorder behaviours and eating disorders are used interchangeably to emphasise women's own perspectives and experiences with eating disorders, and the importance of studying eating disorders even in the absence of a clinical diagnosis.

## Method

To capture rich information from individuals about how diet and fitness apps may affect eating disorder-related behaviours and perceptions, a primarily qualitative research approach was employed. This methodology allowed for users to share their stories and experiences in their own words and emergent themes unlikely to be discovered when using only quantitative approaches. Three data collection methods were used: surveys (demographic and eating disorder symptoms survey), think-aloud exercises and semi-structured interviews.

### Recruitment

In total, 24 participants took part in the study. The focus of this research was university women with eating disorders who use or have used diet and fitness apps in the USA. Participants who were either formally or self-diagnosed were recruited. This was specifically done to include the portion of women who do not seek a professional diagnosis or treatment. Therefore, this study represents users whose needs are largely invisible. This population is important to study because anorexia nervosa, bulimia nervosa and related eating disorder behaviours tend to affect university women,^8^ and diet and fitness app users tend to be younger.^1^ To recruit users, on-campus groups were asked to share information on a campus listserv and fliers were posted to social media. Additionally, paper fliers were posted on bulletin boards on and off campus, such as at local gas stations. Because eating disorders are stigmatised conditions, many people may be wary of being seen getting contact information from fliers. Posting paper fliers in discreet locations, such as on the backs of doors in public restroom stalls where participants could covertly obtain information for the study, was the most successful approach.

### Measures

#### Demographic and eating disorder symptoms survey

The survey contained questions about age, gender, and race/ethnicity, as well as eating disorders and app use. A combination of three well-known measures for assessing the severity of disordered eating and exercise behaviours and attitudes was used, which is similar to Tan et al^17^ and described in Table 1: the Eating Attitudes Test (EAT-26),^18^ the Eating Disorder Examination Questionnaire (EDE-Q 6.0)^19^ and the Clinical Impairment Assessment Questionnaire (CIA 3.0).^23^

**Table 1:** Description of eating disorder symptoms measures

Measure	Description	Interpretation
EAT-26	The EAT-26 is a 26-item self-report questionnaire that assesses symptoms and concerns characteristic of eating disorders on a six-point scale (always to never), using behavioural questions regarding the past 6 months.^18^ It is often used as a first step in a multi-stage screening process and has been useful as a screening tool to assess eating disorder risk. It consists of three subscales – diet, bulimia and food preoccupation, and oral control – that make up an overall score. As part of the EAT-26, participants are also asked to self-report their height, current weight, ideal weight, lowest adult weight and highest adult weight.	For those who score >19 and/or qualify for one or more of the behavioural questions, the recommendation is to see a qualified professional because they are exhibiting symptoms characteristic of eating disorders. Even without the cut-off score, this measurement can be used as a continuous measure of eating disorder symptoms.
EDE-Q 6.0	The EDE-Q 6.0 is a 28-item self-report questionnaire that measures frequency and impact of eating disorder behaviours in the past 28 days that reflect severity of aspects of the psychopathology of eating disorders, using seven-point scales (no days to every day; not at all to markedly), and questions where respondents report the number of times or days they engaged in particular behaviours.^19^ A highly reliable and validated tool, the EDE-Q 6.0 is the most commonly used assessment for eating disorders.^17,20^ It consists of four subscales – restraint, eating concern, shape concern and weight concern – which make up the global score.	Higher scores indicate greater levels of symptoms. Suggested cut-offs range from 2.30 with the occurrence of binge eating and/or excessive exercise in community samples of young women to indicate ‘probable’ eating disorders,^21^ to ≥2.80 for clinical samples.^22^
CIA 3.0	The CIA 3.0 measures the severity of psychosocial impairment from eating disorder features in the past 28 days, on a four-point scale (not at all to a lot).^23^ It is a 16-item measure that focuses on mood, self-perception, cognitive functioning and work performance, which is intended to be taken after a measurement of current eating disorder behaviours (such as the EDE-Q 6.0). It then provides values to assess the severity of psychosocial impairment secondary to eating disorders.	Higher scores indicate greater psychosocial impairment. A score of 16 is suggested as a cut-point for predicting eating disorder case status.

EAT-26, Eating Attitudes Test; EDE-Q 6.0, Eating Disorder Examination Questionnaire; CIA 3.0, Clinical Impairment Assessment Questionnaire.

#### Think-aloud exercise

The think-aloud is a method in which participants speak out loud thoughts that come to mind as they go through a task.^24^ The objective with the think-aloud exercise was to explore participants’ perceptions linked to specific aspects of the app. Participants went through three tasks: setting goals, viewing progress visualisations and using social and community features of the app. As users went through these tasks, they were asked to speak aloud what they were thinking and feeling as they interacted with the app.

#### Semi-structured interview

The purpose of the interviews was to understand participants’ general experience with and perceptions of diet and fitness apps. Participants answered questions regarding why they used diet and fitness apps, the role the app played in their eating disorder behaviours (both positive and negative), unanticipated effects and their reflection on their use over time. At approximately 14 interviews, repetitive themes in the participant responses were apparent and converged into the same points (i.e. data saturation).

### Procedure

Although there were distinct methods of data collection, they occurred during the same session. All sessions began with the demographic and eating disorder symptoms survey. All participants took the demographic survey; five opted not to take the eating disorder symptom survey. Current app users (*n* = 17) then participated in the think-aloud followed by the interview. Former app users (*n* = 7), on the other hand, only participated in the interview after taking the survey. In those cases, participants discussed how they used the app and were asked to recall specific features. Participants were compensated $25 each for approximately 1 h of their time. All but one data collection session took place in person (one was conducted via telephone).

### Ethics

All procedures contributing to this work comply with the ethical standards of the relevant national and institutional committees (Institutional Review Board approval number: STUDY00004634) on working with human participants. Institutional review board approval was obtained from Pennsylvania State University, and written informed consent was obtained from all participants. Materials were reviewed by a mental health professional. Resources were provided to every participant. Participants who currently did not use diet and fitness apps were not asked to interact with apps to avoid potential triggers. A plan was in place to work with participants in seeking support should they need it during or after a session; participants were reminded they could cease the session at any point. Because participants were students at one university, the university's Center for Counseling and Psychological Services was available to participants.

### Data analysis

Excel for MacOS and JASP for MacOS (JASP Team, University of Amsterdam, the Netherlands; see https://jasp-stats.org/) were used to organise and analyse the quantitative data from the demographic survey and eating disorder symptoms measures. Body mass index (BMI) was derived from height and weight data. For those aged ≥20 years, BMI was computed with the United States National Institute of Health calculator (https://www.nhlbi.nih.gov/health/educational/lose_wt/BMI/bmicalc.htm), and for those aged <20 years, BMI was calculated with the Centers for Disease Control and Prevention calculator (https://nccd.cdc.gov/dnpabmi/calculator.aspx). Think-aloud exercises were video and audio recorded, and interviews were audio recorded. In total, the think-aloud exercises and semi-structured interviews were 21 h and 36 min. The think-aloud exercises and interviews were transcribed for a total of 436 pages, and analysed together. The data were analysed by the author, using Braun and Clark's thematic analysis approach,^25^ which included becoming familiar with the data, systematically identifying codes and themes, and defining and naming the common themes found across the entire data-set. Similar discussions and answers were grouped together, and initial codes related to unintended negative consequences were developed. During data collection, the analysis was iteratively performed to refine the themes as more data was collected. The videos and still images were used to better understand specific app content and features to which participants were referring.

## Results

### Participant demographics

Participants were aged 18–23 years, with a mean of 20.63 years. The majority of participants identified as White (non-Hispanic) (*n* = 18), with one from Israel; three identified as Asian, Asian American or Pacific Islander; two identified as multi-racial and one identified as Native American or American Indian. Most participants had not been professionally diagnosed with an eating disorder (*n* = 17), and most reported being in recovery or recovered (*n* = 20). Participants estimated they had an eating disorder anywhere from 2 months to 7 years (mean 34.93 months, s.d. 26.78 months), and most (*n* = 20) felt that their eating disorder began before using diet and fitness apps. The most used app was MyFitnessPal (*n* = 21); however, many of the other apps used had similar features to MyFitnessPal. Participants reported using diet and fitness apps anywhere from 2 months to 8 years (mean 30.21 months, s.d. 30.05).

Participants reported current (mean 22.90, s.d. 3.58), high (mean 24.71, s.d. 3.84), low (mean 19.54, s.d. 3.40) and ideal BMI (mean 21.13, s.d. 2.26). At the time of data collection, most participants were in the healthy range (*n* = 16), followed by overweight (*n* = 2) and obese (*n* = 1). Highest reported BMI for participants was most often in the healthy range (*n* = 14), followed by overweight (*n* = 3) and obese (*n* = 1). Lowest reported BMI most often fell in the underweight (*n* = 8) or healthy range (*n* = 8), followed by overweight (*n* = 2). Most participants reported an ideal weight in the healthy range (*n* = 17), followed by underweight (*n* = 1) and overweight (*n* = 1). Seventeen out of nineteen participants reported their ideal weight as less than their current weight, and only two reported their ideal weight as higher or the same as their current weight.

Sixteen out of nineteen participants answered one or more of the eating disorder questionnaires in a way that suggested eating disorder symptoms. For the EAT-26, the overall mean score was 21.32 (s.d. 10.63), and 15 out of 19 participants exceeded the cut-off point. For the CIA 3.0, the overall mean of all 19 participants did not reach the cut-off point of 16 (mean 14.84, s.d. 10.39); however, nine participants exceeded this threshold. For the EDE-Q 6.0 global score, the overall mean of 2.70 (s.d. 1.04) was between suggested cut-off points.^21,22^ Scores were also compared with the norms of university women, which was computed by taking the norm mean (1.65) and adding 1 s.d. (1.30), to equal 2.95;^26^ ten participants exceeded this threshold. Additional information can be found in Supplementary Table 1 available at https://doi.org/10.1192/bjo.2021.1011.

### Themes

Eight types of unintended negative consequences from using diet and fitness apps emerged, which can be seen in Table 2. These themes focus on the interaction between the user, context and app, and how the design of apps affects attitudes and behaviours. These themes include fixation on numbers, rigid diet, obsession, app dependency, high sense of achievement, extreme negative emotions, motivation from ‘negative’ messages, and excess competition. Although these were common when users’ focus was to lose weight or eat less, these adverse effects were also prevalent when users wanted to gain weight, eat more or focus explicitly on eating disorder recovery. As a result of these unintended negative consequences, some participants reported secondary effects, such as interference with personal relationships, social outings, school and work, as well as increased health issues.

**Table 2:** Emergent themes, definitions and example quotations

Theme	Definition	Example quotations
Fixation on numbers	Developing a fixation on numbers associated with food and exercise, an acute awareness of calories, an altered relationship with food and/or a need for exactness from the quantifications within the app	‘I think it's [logging food and exercise everyday] definitely very triggering because you look at food differently. Like now when I look at food, I see like that's protein, that's fat, that's carbs instead of like that's a chicken breast, that's peanut butter, that's a piece of bread… it's definitely very, very triggering to be tracking it all the time. And especially back then [during my eating disorder], it was like, “Well, that's 100 calories right there, like I need to eat broccoli instead, that's like 35 calories”… It's a number game basically….’ [U06]
		‘I try to get exactly on [the number]… I like having it exactly on… It [the app] made me more OCD [obsessive compulsive disorder] ‘cause I'm like, “I have to hit this number”, basically… making sure I hit those numbers… There was one time my parents wanted to go out to dinner… So, I called the [restaurant] so I could already track it and have it as close as possible. And then my parents get here, and they're like, “Oh, we're going go to [this other restaurant] instead”. And I was literally having anxiety about going. I didn't want to go to dinner. I was like, “No. I already had everything perfectly planned for my day”, and that was probably a bad moment… I feel like eating disorders stem from people trying to be perfect, and with this, you're hitting numbers trying to be perfect, so I think that could be kind of bad’ [U14]
Rigid diet	Developing a strict and rigid diet, including eating the same foods every day and/or developing safe and fear foods through the use of the app's food database, personalised prior meals or the barcode scanner	‘I think another kind of bad thing about it is I eat the same thing almost every single day except for dinner, but I think like just because in my head, I can kind of keep track of the points, and I think that's probably part of it. I'm not going to eat like a lot of new stuff if I have to like kind of go and do the work for it and see how much it is, so I think that kind of makes me eat the same thing every day’ [U08]
		‘I love how it could scan a label… That was my favourite thing in the world… It got to the point where I would never buy something that didn't have a label on it ‘cause I couldn't track it… And I would be very secretive about just having a picture and being able to successfully find it on the app. If I couldn't find it on the app, I wasn't going to eat it ‘cause… It wouldn't have been correct… You start to eat the same things… ’[U17]
Obsession	Becoming obsessed with logging and tracking, which can lead to the development of obsessive thoughts around food and exercise	‘I just think the entire app in general is harmful… For someone like me, it's extremely dangerous. Just everything. Being able to log your calories, ‘cause you become obsessive over taking pictures of labels, you're measuring things, and getting the correct amounts becomes impulsive and just like obsessive. Exercise then, plays a same role in that… I got to be honest: before I started using the app, I felt like my logging wasn't that dangerous. It wasn't that compulsive or that obsessive, I should say…’ [U17]
		‘…I remember, I had that year at least five, six anxiety attacks because I was so anxious about what I'm eating, and I was so nervous about it. And the app said one thing and then the computer said something else, and I just lost my mind… So for me, it emotionally was a bad thing, the app… That's when I was really obsessing, and I would make sure everything is measured to the centimetre, to the ounce… I think it [the app] makes us overthink food, which can lead to obsessing about it… So I think the focus should be way more on health and way less of numbers… I think this [the app] just reinforces the wrong thing’ [U12]
App dependency	Feeling that one needs the app, feeling safe and in control with the app, developing anxiety when not using the app and/or not wanting to cease app use	‘In the moment, I didn't care. I knew it [the app] was harming my brain because I knew it was messing with my head mentally, but I just wanted to keep it because I felt like that was the one thing I could control. Because when you have an eating disorder, that's the one thing you want, is control. And I knew this app gave me control over what my parents wanted me to eat, just in that sense. I never really told them ‘cause I didn't want to lose that control I had. Because being forced to eat a sandwich or being forced to eat, to go see a therapist, I had no control over those, but with the app, I felt like I had control over one part of my life that I really wanted to change’ [U21]
		‘Last summer, I had to delete it [the app]. I deleted it and had to get it back ‘cause I was like, “Oh, my gosh, I need to know what I'm eating”… I literally got anxiety, so I had to get it back… I was like, “Maybe I should just stop tracking and just eat intuitively”. So, that's why I tried deleting it. And then like a few days later, I had to get it back…’ [U14]
High sense of achievement	Feeling extremely rewarded for eating under calorie and nutrient budget, engaging in compensatory behaviours and inputting them on the app, and losing weight; often occurs when receiving positive feedback on the app, such as via green visualisations	‘I definitely would say that if I got to the end of the day… like if on Tuesday, I was a little bit more in the green [on] Wednesday, I'd feel better about it. So it was almost like an accomplishment *per se*… Sustaining it [my eating disorder] would absolutely be seeing that when you're low or you're in the green… You don't even think about green being a good thing but just the colour cues that you associate with rewards… when you're starting to reinforce eating less, eating less, eating less… So I think it's [the app's] very much targeted towards the weight loss rather than fitness, *per se*’ [U19]
		‘I obviously like to be in the green for the calories remaining… This thing, progress bar, I mean, I kind of like, I mean, I used to like to see it really close to that like goal line or even like below, which sounds bad. But because that looked better to me if the bar's lower. So I mean, I guess, maybe that's kind of a problem, but I mean, it kind of made me feel that I was kind of like successful for the week if it was like mostly under the bar, obviously [laughs] even though that's under your calorie thing, which is probably not good… I just kind of wanted to see where I was in my calories for the day, and if I was like under what they allotted me, then I was happy… If I went to this bar and I saw everything was like below the goal, then that would kind of make feel like all right, that was good’ [U04]
Extreme negative emotions	Feeling extreme negative emotions, such as guilt, embarrassment and shame, especially when exceeding one's calorie or nutrient budget or gaining weight; often occurs when receiving negative feedback on the app, such as via red visualisations	‘At the end of the day, if I was still very hungry and I didn't have any calories left, that whole red number… That red number would scare me a lot because I'd be like, “Well, now I can't eat anything, and I'm really hungry, and I can't sleep with an empty stomach”. Then if I ended up eating, I would wake up feeling guilt for going over my intake because I felt like it would get in the way of my goal of losing weight… Once it hit 200 or more, I would get really stressed out, even panic because… I would be ashamed because I felt like I wasted my whole day of when I was fasting ‘cause when I was fasting, it would be a really low goal of calories… So it was just very stressful to deal with the red numbers… The red number would come, and I'd be over my calories, and it just freaks me out all the time. I wouldn't even want to go to school if I knew I ate too much that night or that day before… I feel guilt for what I ate that day ‘cause it's usually something that was high in calories, like a cookie or something. And then that caused that to become a fear food, like dietitians like to call it, a fear food that I try to exclude from my diet because that leads to a red number that embarrasses me’ [U21]
		‘I don't like the colour red. I feel like it's bad, and it would always be like a frowny face, like bad, like you didn't do what you're supposed to today, and I was like, “I know, I know I didn't”… I think they definitely need to be not as like strongly represented. Like if you're 1 calorie over, it's like, “Ok, like no big deal”. It should be like a range, you know what I'm saying? One calorie over is different than being like 400 calories over, and I think it definitely gave me the wrong perception and made me kind of go like the other way especially like when all my things were red in [my] app, I was like, “Ok, well, then this makes me definitely not want to eat for like 3 days after seeing that”’ [U05]
Motivation from ‘negative’ messages	Feeling motivated by ‘warning’ messages usually intended to curb unhealthy behaviours, such as feedback that states low weight or low calorie intake	‘…I was under-eating, so they [the app] would show me, you would be 90 pounds in a month or something if you kept on eating like this… I would just under-eat more to make that happen faster… So, I used to exercise 400 calories, then I would just skip lunch, I would eat dinner… Over here it would be 500 remaining or something. And at that point it would be, “Ok, so you're going to be 95 pounds if you kept on eating like this in 2 weeks”. So that was more of a motivation, I think… Because you're trying to lose so much weight, and you're like, “If you keep on under-eating, you're going to be 98 pounds”, which is exactly what you want to be at that point… It's not a warning…’ [U22]
		‘If you click this “Complete Diary”… So it tells you, “If every day were like today, you would weigh this amount”, which [laughs] it's like I have such mixed feelings about it because like it can be motivating, but also it can be really triggering… like someone with an eating disorder is like, “Yeah, yeah, you're right; oh my god, I can weigh less than that in 5 weeks if I eat less”… When you're in the middle of your eating disorder, you think this is motivational, but when you look back on it, it's like, that's horrible [laughs], like that's really horrible’ [U06]
Excess competition	Making calorie consumption, expenditure and weight loss a game by trying to beat the app or self; often achieved by netting fewer calories each day and/or being under budget	‘It was kind of like a game to beat the calories, kind of. So one day I had a 0, maybe it was like a negative calorie. I was like, “Oh, wow, like look at me, like that's cool!”…Just because like you can visualise what you're eating, so the more you don't eat, it's like, “Oh, I beat the app!”… I definitely wanted to beat the calories they gave me. I feel like that kind of does start an eating behaviour where you don't want to eat anything… Like especially ‘cause they give you a calorie limit. I know when I was under the calorie limit, I was like, “Ok, I won today”… I was like, wait a second, the app kind of like made it a game for me to like not eat much’ [U07]
		‘It just became this weird competition thing with it [the app]… I would just be like, “I need to be lower than what it was before”. [laughs] I don't know… It just always had to be less than the day before in the food and the weight and everything… Because then, if I wasn't, then I was like a failure ‘cause that was what the eating disorder thoughts were telling me’ [U13]

Participants discussed developing a fixation on numbers, fuelled heavily by the app's quantification, which worsened their eating disorder behaviours and changed their relationship with food. Having used the apps so much, many participants reported already knowing the calorie content of every food they ate before logging it. Participants also explained that they tended to eat the same foods each day because they knew the calorie content and could mitigate any unknowns about what they were consuming (even if they abandoned the app). The app also fed into the concept of fear foods and safe foods, where users would only buy and track foods if they were aware of their calorie content (e.g. in their personal app database or foods that had a barcode).

They described becoming obsessed with logging their food intake, and developing obsessive thoughts around food and exercise that sometimes interfered with schoolwork. For example, some participants used the app to log all their meals in advance, which acted to strictly control their consumption. Some also described developing a dependency on these apps. Many participants discussed how they needed the app and became very anxious when they stopped using it; they sometimes redownloaded the app to relieve their anxiety. One participant described how uncomfortable she was when she went to a clinician who wanted her to explore the idea of not using her physical activity tracker (Fitbit).

A number of participants described the role of green progress visualisations, which users see when they have remaining calories on MyFitnessPal and similar apps. Many expressed feeling rewarded when viewing this feedback, as it signalled they were consuming less than their allotted calories. On the other hand, participants felt guilt, embarrassment and shame over exceeding their calorie budget and being shown red visualisations in response. The extent to which they exceeded their budget affected participants differently. Some expressed that they felt badly regardless of how much they went over their budget, whereas others explained how they felt worse the higher their calorie number exceeded their budget. Many participants also described being in an unhealthy competition with themselves and with the app to eat less and less each day, because the app ‘gamified’ eating, exercise and tracking.

Although there are some features in diet and fitness apps that attempt to curb maladaptive eating and exercise behaviours, participants explained that these did not work as intended. For example, MyFitnessPal has a feature called ‘Complete Diary,’ which is a button that allows users to tell the app they are finished logging food, exercise and weight for the day. Once clicked, either a warning message or weight projection appears. Many participants found both types of messages to be motivating to continue to lose weight regardless of the content or context of the message.

## Discussion

Unintended negative consequences are prominent regardless of where users are in their journey (e.g. recovery or not). This is a result of the design of diet and fitness apps, the individual and their context. This section first discusses implications for educators and clinicians, and then critically examines the design of diet and fitness apps and offers suggestions for improvement.

### Implications for clinicians and educators

Understanding the unintended consequences can be useful for psychiatrists, psychologists and other mental health experts, as well as general practice clinicians, to aid in the diagnosis and treatment of eating disorders. Especially in college and university settings, healthcare professionals should be aware of and engage in discussions about the use and potential downsides of diet and fitness apps. Educators should also be privy to possible unintended negative effects to prevent triggering or exacerbating maladaptive eating and exercise behaviours. By encouraging or even requiring the use of digital food and physical activity tracking as part of nutrition courses and ‘healthy’ university initiatives (e.g. https://www.usatoday.com/story/college/2016/01/19/oklahoma-college-tracks-students-fitness-with-fitbits/37410983/), educators may unknowingly exacerbate eating disorder-related issues, especially among university women. Therefore, great caution should be exercised when considering promoting diet and fitness apps, especially in these settings. As always, it is important to remember that app users and app use exist in a larger context, where societal norms and external pressures influence the effects of these tools.

### Rethinking diet and fitness app design

The design of diet and fitness apps may partially contribute to unintended negative consequences, which are related to three major areas: the quantified self movement, our conception of appropriate usage, and visual cues and feedback. Table 3 outlines how these findings relate to app design, to help us understand where we can make improvements to minimise unintended negative consequences and focus more on promoting healthy behaviours. However, it is important to note that although small changes may have some positive impact, this work highlights the need to change how we think about health promotion in digital tools by focusing on the mental health needs of users and the interplay between mental and physical health. A more holistic and personalised approach is consistent with prior literature on supporting the needs of people with eating disorders.^27^

**Table 3:** Summary of suggestions to address diet and fitness app issues

Area	Problem	Related unintended negative consequences	Suggestions
Quantified self movement	Overabundance of quantification despite the fact that not all aspects of health can easily be quantified Some quantifications are not good health indicators Too much of a number focus can trigger and exacerbate eating disorder behaviours	Fixation on numbers Rigid diet Obsession App dependency High sense of achievement Extreme negative emotions Motivation from negative messages Excess competition	Work meaningfully with people with eating disorders during all phases of designing these tools Find new ways to acquire user needs and non-numeric yet quick and easy methods for tracking behaviours Consult expert recommendations about healthy eating and exercise during design process Support healthy eating patterns, food variety, portion control, shifting to better food choices and various eating contexts Change exercise tracking to focus on performance and enjoyment rather than calorie expenditure Incorporate qualitative components to assess other aspects of health
Conception of appropriate usage	Push users to log consistently over long periods of time Tend to view breaks and abandonment as negative Encouraging overuse can trigger and exacerbate eating disorder behaviours	Obsession App dependency	Reduce reminders to log daily Encourage breaks Rethink what app engagement is and reward users for engagement that is not actively logging or viewing numbers
Visual cues and feedback	Try to use visualisations to motivate users but do not fully understand their effects Visual cues do not always match users’ goals Warnings have opposite of intended effect or are avoided Visualisations and messages can trigger and exacerbate eating disorder behaviours	High sense of achievement Extreme negative emotions Motivation from negative messages Excess competition	Study effects of design and warnings more thoroughly and on different users at different times Develop more nuanced design visualisations that better coincide with intended messages and users’ goals Rethink unhealthy eating and exercise pattern thresholds for showing feedback

#### Quantified self movement: moving beyond numbers

The quantified self is reflected in diet and fitness apps’ heavy focus on numbers. Although self-tracking numeric data has benefits,^28–30^ findings cast light on issues with the quantification and tracking of behaviours related to diet and exercise, especially for those with a history of eating disorders, which has been supported by other literature.^6,31^ Users with eating disorder behaviours develop a fixation on numbers and a rigid diet partly because of diet and fitness apps’ heavy focus on numbers, as well as features such as barcode scanners, which are aimed at reducing user burden but actually encourage eating pre-packaged and fast foods,^32^ which often are not the healthiest options. Because food, exercise and weight are quantified and goals are numerically driven, users become overly preoccupied with numbers, and food begins to be viewed as its caloric and macronutrient content.

Although the quantified self movement has its merits, it is clear that using numbers as indicators of health has its limitations and feeds into the need for control, which is a hallmark of eating disorders. To begin to reduce unintended negative consequences, designers, developers and researchers need to focus less attention on quantifying food, weight and exercise. Instead, understanding what a healthy lifestyle is and finding ways to promote that with technology is imperative. For example, rather than focus mostly on calories, apps should be designed to help users develop a positive relationship with food and their body, as well as healthy eating patterns that include fruits, vegetables, protein, dairy, grains and oils; focus on food variety, nutrient density and amount/portion sizes; help limit added sugars and saturated fats, and reduce sodium intake; find ways to help people shift to healthier options and assist healthy eating in various settings (home, work, school, restaurants, etc).

For physical activity, the focus should be less on exercise's relationship to calories and more on how much exercise, what types, ability to perform, enjoyment and its relationship to positive mental health. Studies have shown that exercising for enjoyment rather than appearance is correlated with low self-objectification, low body dissatisfaction and less disordered eating.^33^ By focusing on exercise as something enjoyable and healthy, the focus will be less on exercise as a means to lose weight or look ‘better’, and thus improve overall mental health. Apps should also adapt to users’ personal contexts and needs around physical activity and healthy eating, as well as acknowledge systemic barriers and the role of trauma. Because customisation may be crucial for supporting users’ needs, more sensor-based and passive tracking are being explored.^34^ However, caution must be exercised, as automated detection often reproduces biases and existing norms, exacerbating inequities, which can worsen mental health.

Other important aspects of health not easily captured in many current diet and fitness apps include positive body image, mental health and bodily functioning. For example, does a user feel good in their clothes? How is their self-esteem, emotion regulation, concentration, etc? Are they depressed, anxious, etc? Are they experiencing any pain or discomfort? Are they less tired throughout the day, and do they have improved sleep? All these things are important aspects of health. Even for users whose weight loss is a healthy goal, these factors may influence their needs and ability to lose weight, which means supporting these needs can positively affect all users.

#### Conception of appropriate usage: encouraging less logging (in some cases)

The quantified self movement coupled with our conception of appropriate app usage can lead to an obsession (about logging, food, weight and exercise) and the development of an app dependency, which is partly fuelled by how much and how often designers, developers and researchers think people should use these types of digital tools. To promote consistent and long-term use, many apps contain reminders to log and gamified aspects (e.g. streaks). This, coupled with the quantification, leads to users becoming obsessed with logging, which is in line with prior research.^6,32^ However, contrary to some research,^32^ users with eating disorder behaviours do not really ‘lose the habit’ of logging, because they feel the need to have control over their food and body. Despite numerous studies aiming to reduce app abandonment,^32,35,36^ abandonment is not always negative. In fact, for users with eating disorder behaviours, taking a break from apps can be beneficial.^7^ Taking time off from apps can help users learn to listen to their body's signals of hunger and fullness and decrease their dependency on apps, which is important if we wish to promote health. Therefore, reducing logging reminders and encouraging breaks may be beneficial. Ways to reward users for engaging with apps without viewing quantified behaviours or actively logging (e.g. providing an alternative app view during break periods) could be explored.

Moreover, we need to ask ourselves: what role should these apps play in users’ lives? Are they meant to be used every day throughout a person's life or are there more finite periods? How do we determine a success versus a failure (and should we impose a viewpoint of ‘success’ or allow users to choose)? We have to stop pushing an ideal, universal use and start understanding how people actually use these technologies ‘in the wild’, and how their needs change over time. Then we can design around their natural patterns of use, be more adaptive and flexible, and acknowledge different situations and contexts. Although app vendors want users to use their technology long term, we also must understand that this is not appropriate for all users and may even be harmful for some.

#### Visual cues and feedback: investigating effects more thoroughly

Findings show that app visualisations and feedback, such as coloured visualisations and messages, can unintentionally contribute to unhealthy behaviours. Instead of promoting healthy behaviour change, red and green visualisations in combination with the focus on numbers often result in users feeling a high sense of achievement when being under their calorie budget and extreme negative emotions when being over their budget, which has been seen in other research.^6^ These colours were likely chosen because of the connotations they already have in some societal contexts. However, these effects in the context of diet and fitness apps are not well studied. Studying these effects is crucial, given that the effects of colour choice can vary from context to context.^37–39^ Thus, we need to examine the effects of colours on users, and find ways to balance emotion response and behaviour change strategies.

The rewards and punishments users get from diet and fitness apps through these visualisations and the focus on the quantified self often promote excess competition. Although many apps want to encourage competition, users with eating disorder behaviours often develop unhealthy competitive behaviours. Not only do these visualisations instil a sense of reward in punishment in users, but they also tend to be very limited. For instance, at the time of this study, in MyFitnessPal, users see the red number regardless of whether they exceed their daily allotment by 1 or 1000 calories, which does not make sense if the focus of these apps is promoting health. Therefore, we need to develop more nuanced visualisations to motivate users without negatively affecting them.

Users also felt motivation from (what are intended to be) ‘negative’ messages and visual cues. For example, the ‘Complete Diary’ function in MyFitnessPal is meant to motivate users in the appropriate context and provide a warning to deter unhealthy habits. In many instances, users felt both messages motivated them to continue unhealthy behaviours regardless of the content, suggesting that more research is needed to understand how warnings and other feedback messages influence user perceptions and behaviours. One of the issues lies in the threshold that is used to determine with what feedback is presented. Although these algorithms are proprietary to MyFitnessPal, at the time of this study, MyFitnessPal seems to use a baseline of 1000 calories consumed to determine which message the app shows. If users do not hit this threshold, then they are shown the ‘Based on your total calories consumed for today, you are likely not eating enough’ message. If users consume over 1000 calories, then the app presents ‘If every day were like today, you would weigh X pounds in X weeks’ message. This occurs regardless of how many calories users have remaining. Thus, more research is needed to understand the appropriate thresholds to use to provide different feedback based on users’ needs.

Further, precautions such as warnings should not focus on taking away someone's agency or labelling someone or their behaviours as ‘bad.’ There is a tendency to do this with eating disorder-related behaviours, which can increase stigma and reinforce negative emotions. Rather than adding these types of features, users and potential users from a variety of backgrounds should be more meaningfully involved in all aspects of the design process in a way that honours their lived experiences as expertise, and have the power to inform design decisions within these apps.

### Limitations

First, the sample comprised a small subset of rather homogenous users. Thus, it is likely that not all consequences and perceptions are represented in this work. Future research should include more users from a variety of races, ethnicities, cultures, genders, ages and types of conditions. Second, BMI has a number of problems and limitations. It was used in this study as way to provide additional information only, not to advocate for its blanket use to denote health or diagnose/treat eating disorders. Third, unfortunately, normative clinical data that have similar contexts and participants are not easily available for all measures. In general, the means reported in clinical samples for the EDE-Q 6.0 and CIA 3.0, (e.g. Dahlgren et al^40^) are higher than the present study; however, it is important to note that participants in this study were often recollecting past experiences with eating disorder behaviours, and many reported being in recovery currently. Thus, it is possible that eating disorder symptom scores at the time of the study were lower than they would have been if the study had occurred during what participants described as the worst points of their eating disorders. The findings suggest that specific design choices are problematic for some users. However, these design features and choices themselves were not tested. Research could benefit from experimental testing of these designs, as well as participatory and community-driven design of diet and fitness apps.

In conclusion, the use of diet and fitness apps by women with eating disorder behaviours is likely more common than many realise, given the rates of dieting and weight loss among healthy weight and underweight women.^41,42^ This work identifies problematic aspects of design and design suggestions, as well as implications for clinicians and educators. Although this study focuses on users with a history of eating disorders, redesigning apps to focus on health is beneficial to all users. Ultimately, this research emphasises the need for a fundamental shift toward a more holistic, personalised approach to health and how it is represented in digital tools.

## Data Availability

The data that support the findings of this study may be available upon reasonable request from the corresponding author, E.V.E. Participant privacy and consent is of utmost importance. The data are not publicly available due to their containing information that could compromise the privacy of research participants.

## Supplementary material

For supplementary material accompanying this paper visit http://dx.doi.org/10.1192/bjo.2021.1011. click here to view supplementary material
