# Supplementary material for: Effects of diet and fitness apps on eating disorder behaviours: qualitative study
Source: BJPsych Open. 2021 Sep 24;7(5):e176. doi: 10.1192/bjo.2021.1011 (PMC8485346; doi:10.1192/bjo.2021.1011)
Supplement: Supplementary file 1 [file S2056472421010115sup001.docx]

**Supplementary Material**

**Supplementary Material Table 1. Participants' BMI and eating disorder symptom scores.**

| **ID** | **Current BMI** | **High BMI** | **Low BMI** | **Ideal BMI** | **EAT-26 Score** | ***EAT-26 Behavioral Questions*** | **EDE-Q 6.0 Global Score** | **CIA Score** |
| --- | --- | --- | --- | --- | --- | --- | --- | --- |
| U01 | 18.70 (13^th^ percentile) (healthy) | 21.60 (50^th^ percentile) (healthy) | 18.00 (6^th^ percentile) (healthy) | 16.20 (< 1^st^ percentile) (under) | **30.00** | **Yes: C E** | **2.96** | **24.00** |
| U02 | -- | -- | -- | -- | -- | -- | -- | -- |
| U03 | -- | -- | -- | -- | -- | -- | -- | -- |
| U04 | -- | -- | -- | -- | -- | -- | -- | -- |
| U05 | -- | -- | -- | -- | -- | -- | -- | -- |
| U06 | 21.10 | 23.20 | 16.40 | 20.40 | 2.00 | No | 0.51 | 3.00 |
| U07 | -- | -- | -- | -- | -- | -- | -- | -- |
| U08 | 22.00 | 23.40 | 20.90 | 21.10 | **24.00** | **Yes: B C** | 2.78 | **16.00** |
| U09 | 22.50 | 23.30 | 18.50 | 20.90 | **31.00** | **Yes: A** | 2.71 | 10.00 |
| U10 | 27.90 | 30.10 | 25.80 | 24.40 | **21.00** | **Yes: A B C** | **3.01** | 7.00 |
| U11 | 19.50 | 23.50 | 18.90 | 18.90 | **25.00** | **Yes: A** | 2.35 | **22.00** |
| U12 | 21.60 | 22.60 | 20.80 | 20.70 | **29.00** | **Yes: A B** | **3.38** | **20.00** |
| U13 | 23.50 | 23.50 | 15.70 | 20.40 | 8.00 | **Yes: B** | 2.37 | 5.00 |
| U14 | 20.40 | 21.80 | 17.80 | 20.10 | 14.00 | No | 1.44 | 4.00 |
| U15 | 23.80 | 26.60 | 18.30 | 21.60 | 12.00 | No | **3.44** | **16.00** |
| U16 | 29.10 (93^rd^ percentile) (over) | 31.10 (95^th^ percentile) (obese) | 27.30 (90^th^ percentile) (over) | 23.30 (71^st^ percentile) (healthy) | **47.00** | **Yes: A C** | **3.93** | **36.00** |
| U17 | 20.10 | 22.30 | 16.70 | 19.70 | **25.00** | **Yes: C D E** | **3.06** | 11.00 |
| U18 | 22.10 | 23.20 | 20.30 | 21.10 | **23.00** | **Yes: A C** | **3.27** | **17.00** |
| U19 | 20.00 (29^th^ percentile) (healthy) | 23.40 (69^th^ percentile) (healthy) | 17.60 (4^th^ percentile) (under) | 20.20 (31^st^ percentile) (healthy) | **24.00** | **Yes: A** | 1.93 | 13.00 |
| U20 | 24.80 | 26.60 | 15.80 | 23.90 | 5.00 | No | 0.38 | 1.00 |
| U21 | 22.30 (62^nd^ percentile) (healthy) | 23.00 (68^th^ percentile) (healthy) | 19.20 (22^nd^ percentile) (healthy) | 21.50 (52^nd^ percentile) (healthy) | **25.00** | **Yes: B** | **3.71** | **25.00** |
| U22 | 19.20 | 19.50 | 17.10 | 19.20 | **29.00** | No | **2.99** | 4.00 |
| U23 | 24.20 (77^th^ percentile) (healthy) | 25.20 (82^nd^ percentile) (healthy) | 20.30 (37^th^ percentile) (healthy) | 21.30 (50^th^ percentile) (healthy) | 17.00 | **Yes: A B** | 2.69 | 12.00 |
| U24 | 32.30 | 35.50 | 25.80 | 26.60 | 14.00 | **Yes: B C** | **4.35** | **36.00** |
| ***Key:***   \| Underweight \| Healthy weight \| Overweight \| Obese \| \| --- \| --- \| --- \| --- \| \| Bold indicates hitting the cut-off point (EAT-26 and CIA) or exceeding the norm (1.65) + 1 SD (1.30) = 2.95 (EDE-Q global score) (28) \| \| \| \| | | | | | | | | |
